# Survey of patient satisfaction after bilateral cataract surgery

**DOI:** 10.22336/rjo.2022.9

**Published:** 2022

**Authors:** Kozma Kinga, Horváth Karin Ursula

**Affiliations:** * Department of Ophthalmology, “George Emil Palade” University of Medicine, Pharmacy, Science and Technology, Târgu Mureș, Romania

**Keywords:** binocular cataract, pain, asymptomatic

## Abstract

**Objective:** Postoperative assessment of patients diagnosed with binocular cataract, who underwent two phacoemulsification treatments at different times, in terms of subjective experience of the two procedures.

**Material and method:** The investigation is a prospective study based on patients of Ophthalmology Clinic of Mária Street in Budapest and the Ophthalmology Department in Târgu Mureş, between January 2020 and April 2021. After surgery, the patients were surveyed using questionnaires. A total of 53 responses from patients who had undergone cataract removal in both eyes were processed. Data was processed using Microsoft Office Excel and GraphPad Prism 8.0.1.

**Results:** A statistically significant difference (p = 0.0008) in pain was found between the two interventions, with patients reporting greater pain after the second surgery compared to the first treatment. The subjective increase of visual acuity was significantly different (p=0.0156) between the two surgeries. After the first treatment, 37 patients affirmed that their visual acuity met their expectations, but, by the second operation, this had dropped to 31. There was also a statistically significant difference between the individually perceived duration of the two treatments (p=0.0013), with the most frequently reported duration of the first phacoemulsification treatment being 10 minutes (43.4%), and the second eye treatment being 20 minutes (37.7%). Assessing the asymptomaticity, a significant difference (p = 0.009) was registered between the two treatments, the asymptomatic reduction for the second operation being decreased by 28.1%.

**Conclusion:** Patients treated for binocular cataract had significantly worse subjective symptoms during the second treatment.

## Introduction

The lens is an ellipsoidal part of the globe of the eye, made up of crystal-clear elements, located in the posterior chamber, between the iris and the vitreous body. It is the largest protein-rich tissue in our body, with a protein content of about 35%. It does not contain blood vessels or nerves, and derives its nutrients by diffusion from the surrounding aqueous humour. The epithelial layer is on its anterior surface, concentrates the potassium and releases the sodium, the ratio of the two in the lens being nearly 10/ 1 [**1**,**2**].

When the ratio of ions (potassium/ sodium) inside the lens is disrupted (reduced), the increase in sodium concentration causes it to attract water, which can lead to cataracts [**1**].

“Cataracta” is a Greek word meaning waterfall. It is the most common disease of the lens. The accumulation of elements in its state - potassium, calcium, lipids, phosphorus - reduces or eliminates its transparency, leading to partial or total opacity [**1**,**2**]. The most common symptom is a loss of visual acuity, but depending on the anatomical location of the opacity, photophobia, monocular diplopia, myopia, color vision changes may occur. Diagnosis is made after pupil dilation, with slit-lamp examination, and the lens in the pupil area appears greyed out [**3**].

Cataracts are a significant cause of blindness and vision loss, affecting millions of people worldwide, and are on the rise globally. Despite the widespread use of phacoemulsification in developed countries, 20 million people are blind to the presence of bilateral cataracts [**4**].

Because of their congenital origin, cataracts are a feature of many dermatological pathologies [**1**].

Cataracts can be divided into: 

1. Primary cataracta: 

1.1. Congenital cataracta: an intrauterine pathological process, the result of genetical damage [**1**].

1.2. Juvenile cataracts: can be diagnosed in the first decade [**5**].

1.3. Presenilis cataracta: appears before the age of 45 [**5**].

1.4. Senilis cataracta: affects older age group.

2. Consecutive cataracta: develops because of other eye diseases [**6**].

3. Cataract in systemic diseases: most common in endocrine diseases [**7**].

Many attempts have been made to treat cataracts conservatively, but so far without success. Medication treatment includes vitamins, hormone-based supplements, essential amino acids. However, these are not effective in treating cataracts, they can only slow down the progression. The only treatment is the surgical procedure: removal of the grayed-out lens [**1**,**2**].

The signs, symptoms and location determine the indication for surgery. The indication for surgery depends largely on the stage of the cataract, the speed of progression, associated diseases and, most importantly, the visual acuity, to what extent it affects the ability to carry out everyday tasks. The social and economic impact of cataract surgery is significant. In the first year after surgery, it increases the economic efficiency of individuals by 1500% compared to the financial value of treatment. One study has shown a relation between visual acuity loss and cognitive decay. Cataract surgery progressively improves cognitive function [**2**,**3**,**4**,**8**].

Today, the most common treatment method is phacoemulsification using ultrasound, which is characterized by a small incision, rapid recovery, anaesthesia of the globe of the eye with drops and few complications [**1**,**9**].

## Objectives

The objective of the investigation was to present the postoperative assessment of patients diagnosed with binocular cataracts, who underwent phacoemulsification treatment at two different times in terms of the subjective experience of the two interventions, as patients’ complaints and dissatisfaction with the second operation could be observed in postoperative follow-up examinations.

## Materials and methods

The research was a prospective study, based on patients of the Ophthalmology Clinic of Mária Street in Budapest and the Ophthalmology Department of Târgu Mureș, between January 2020 and April 2021. After surgery, the patients were surveyed using questionnaires. A total of 53 responses from patients who had undergone cataract removal (phacoemulsification) in both eyes were processed. Based on gender segregation, 22 men and 31 women were surveyed. The surgical technique was the same, the globe of the eye was anaesthetized using lidocaine drops, and the same type of intraocular lens were implanted in both eyes of one patient. This study adhered to the Declaration of Helsinki.

Microsoft Excel and GraphPad Prism 8.0.1 were used to record, process, compare and evaluate the data obtained from the questionnaires. Statistical significance was considered for p<0.05.

## Results

A total of 53 patients diagnosed with binocular cataracts and treated with phacoemulsification, were evaluated. The most common age range for cataracts was 70-79 years, with the youngest patient being aged 32 and the oldest 88.

Only 9% of patients were diagnosed with cataracts at a general eye examination, suggesting that only a small proportion of the population had regular eye examinations, often leaving undiagnosed diseases that, with appropriate therapy, could reduce the risk of blindness. 91% of patients operated with cataracts consulted an ophthalmologist with a complaint of visual acuity loss or other eye problems.

Using a scale of one to five, we investigated the level of the patients’ concern at the time of cataract diagnosis. Grades increased as follows: no concern (1)→slight (2)→medium (3)→strong (4)→very strong (5). 34% of patients (18 patients) had little concern when the disease was diagnosed, and 32% (17 patients) said they had no anxiety at all. 19% were moderately concerned (10 patients), 9% were concerned (5 patients) and 6% (3 patients) were very concerned. They were most afraid of hospital assigning, the use of injections for anaesthesia, and surgical steps.

We surveyed patients who underwent binocular cataract removal to see whether, in their personal experience, overall, from the preparatory steps to discarding the hospital, the two procedures were equally well managed. It was observed that 28.3% of patients reported that the steps of their two cataract procedures did not match. 

Following, some of the most important points were covered, looking at the details of how this difference was affected, and statistically comparing the differences between the two operations.

We asked patients how satisfied they were with the amount and quality of information provided by their doctor during the two cataract surgeries. Using a scale of one to five (1-not at all satisfied, 5-fully satisfied), no statistically significant difference (p=0.2500) was found between the first and second operations in the information patients received about their treatment. 56% of patients were completely satisfied with the information they received about the first cataract removal procedure, and 56% felt the same way about the second procedure. 

**Fig. 1:**
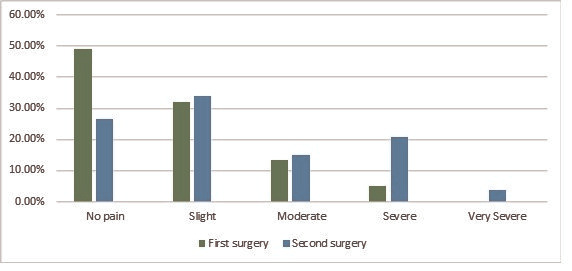
Subjective perception of pain

Our study assessed the subjective perception of pain intensity of the two operations and compared them. Using a scale of one to five (no pain [1]→slight [2]→moderate [3]→severe [4]→very severe pain [5]), patients reported their perceived pain during cataract surgery. There was a significant difference between the two treatments (p=0.0008), patients reporting statistically significant increases in pain during the second operation compared to the first treatment. During the first procedure, 49.1% of patients said they felt no pain at all, but this dropped to 26.4% by the second cataract surgery. Almost the same number of patients ticked the second and third levels of the scale. During the first treatment, the fourth level of pain was 5.7%, the highest level not being mentioned at all. By the second eye operation, this “strength” increased to 20.8% and 3.8% respectively (see **Fig. 1**).

Using Wilcoxon’s measure unit, we examined the degree of subjective visual acuity improvement in patients undergoing surgery, using a scale of one to five, the scores representing an increasing quality of the vision. The test showed a significant difference in the subjective strength of visual acuity improvement between the two operations (p=0.0156). An increase in vision could be observed in all patients after both operations, which was correct, since the aim of the treatment was to regain the quality of visual acuity needed for everyday activities, with the right intervention. Thus, no patient claimed to have experienced “no or little” improvement in visual acuity. However, there was a change at the expense of the second operation. After the first cataract removal, 69.8% of patients experienced the expected results in terms of vision. After the second ophthalmic treatment, only 58.5% indicated that their need was met, where the third grade on the scale, “moderate” vision improvement, showed a numerical increase compared to the first treatment.

Our survey included the patients’ knowledge of the tools and methods used in their treatment. No statistically significant difference was observed between responses to interventions (p=0.6250). In both cases, 47.2% of patients believed that their cataracts were removed using LASER, with only a small proportion, 13.2% being aware that their treatments were performed by ultrasound.

Although the anaesthesia of the conjunctiva and cornea during cataract removal was exclusively by eye drops, 22.6% of patients reported anaesthesia by injection for the first operation and 15.1% for the second. 

We examined the subjectively perceived duration of the two treatments among patients. A statistically significant difference was found between the individually perceived duration of the two cataract removals (p=0.0013), with the second surgery being perceived as the longer one. The most commonly perceived duration of the first intervention was 10 minutes, with 43.4% of patients indicating this time range. 20 minutes showed a frequency of 35.8% and half an hour a frequency of 20.8%. No one noticed the three quarters of an hour. For the treatment of the other eye, 10 minutes fell to 30.2%, with 20 minutes being the most common (37.7%). The rate of half an hour also increased (24.5%), and three quarters of an hour was also among the responses (7.5%).

Post-operative eye examinations showed that patients who underwent a second cataract removal were more likely to complain of local discomfort and disturbance. Using Wilcoxon’s measure unit, in which symptoms were assigned numerically (1 - tearing, 2 - sandy sensation/ feeling of foreign body, 3 - burning sensation, 4 - itching sensation, 5 - pain, 6 - pressure, 7 - no symptoms), we found a significant difference between postoperative complaints (p=0.0099). After the first cataract removal, 65.4% of patients experienced no symptoms, and after the second treatment, this was almost halved, 37.3% of patients being asymptomatic. After the procedures, some patients had more than one type of complaint at the same time. Five patients reported two postoperative symptoms during the first operation, eight patients reported two postoperative symptoms after the second operation and one patient reported three postoperative symptoms. The most common were the feeling of a foreign body/ sandy sensation (17.3%/ 25.5%) and tearing (13.5%/ 23.5%). The burning sensation and pain in the eyes also showed an upward trend with 15.7% and 2% respectively. The pressure type perception of bulbus occurred only after the second phacoemulsification procedure.

Finally, the questionnaire was used to assess which phacoemulsification treatment was more stressful for patients overall, from diagnosis to post-operative day’s inspection. Based on the responses of the subjects in our study, 58.5% found the two eye procedures equally exhausting, while 35.8% found the second procedure more difficult.

## Discussion

A 2019 study found that anxiety levels were significantly higher before and during the first cataract surgery than during the second treatment (p=0.001) [**11**]. A similar result was reported in another study, which found a correlation between reduced blood pressure, heart rate and increased pain [**10**].

There are studies in literature that compare the pain levels of bilateral cataract surgery. A 2019 meta-analysis of 8 studies concluded that the pain of first eye cataract treatment under local anaesthesia was significantly (p<0.00001) less than second eye surgery [**11**]. We found a similar result, the patients who underwent binocular cataract removal experiencing greater pain during the second procedure.

A 2018 survey of patients over 55 years of age with normal cognitive function showed that there was a significant reduction in falls after cataract surgery due to a qualitative improvement in binocular visual acuity (IRR = 5.488, 95%CI = 1.191-25.282, p=0.029). The increase in visual acuity was also confirmed in our study. Literature suggests that this also increases the frequency of outdoor activities [**12**].

The difference in subjectively perceived duration of treatments showed similar results in literature. A survey of 466 patients who underwent bilateral cataract treatment concluded that the estimated time of the surgery was significantly longer during the second treatment (p=0.001) [**13**]. 

A study in literature found a difference in exophthalmia sensitivity, which was significantly confirmed (p<0.05) after the two cataract surgeries, but no demonstrable difference was found for photophobia (p=0.555) [**14**]. In our study, a proportional increase in exophthalmia sensitivity was observed after the second cataract surgery. 

By elaborating our survey, we could state that 35.8% of patients had a more difficult time with the administrative, surgical steps of the second cataract removal. There was also evidence from literature that there was a statistically significant increase in patients’ memories of their second cataract intraoperative surgery. 63.3% could recall more events from the second treatment, compared to only 8.4% from the first intervention.

## Conclusion

Our study suggested that: 

• the willingness of the population to participate in general eye examinations was very low; 

• the level of anxiety was higher during the first operation; 

• patients were satisfied with the information provided on cataract treatment; 

• patients’ intraoperative pain feeling was higher after the second operation, which might have been associated with lower levels of anxiety;

• greater improvement in subjective visual acuity after the first treatment; 

• patients reported the use of intraocular operative injections despite anaesthesia being given only by drops; 

• a high proportion of patients considered the use of LASER a surgical method. 

In order to improve the quantity and quality of the above conclusions, it is necessary to consult before a cataract treatment. The nursing staff, with the involvement of the attending physician, should support the patient preoperatively; sedatives and analgesics may be given to help relieve unpleasant symptoms. Enlightenment about the intervention is an important part of the preparatory step. 

**Conflict of Interest statement**

The authors state no conflict of interest. 

**Informed Consent and Human and Animal Rights statement**

Informed consent has been obtained from all individuals included in this study.

**Authorization for the use of human subjects**

Ethical approval: The research related to human use complies with all the relevant national regulations, institutional policies, is in accordance with the tenets of the Helsinki Declaration, and has been approved by the review board of “George Emil Palade” University of Medicine, Pharmacy, Science and Technology, Târgu Mureș, Romania.

**Acknowledgements**

All authors contributed substantially to this manuscript. The authors acknowledge the technical support from “George Emil Palade” University of Medicine, Pharmacy, Science and Technology, Târgu Mureș, Romania with the Department of Ophthalmology. We would also like to thank all the subjects who participated in this study.

**Sources of Funding**

No source of funding to declare.

**Disclosures**

None.
